# Incidental Diagnosis of Normal Pressure Hydrocephalus in Elderly Patients Presenting With Acute Neurological Illnesses: A Report of Two Cases

**DOI:** 10.7759/cureus.93025

**Published:** 2025-09-23

**Authors:** Kyaw Z Aung, Naw Eh Law Saw, Ei Ei Cho, Cherry Myint, Han Thant Thant

**Affiliations:** 1 Internal Medicine Department, Kulhudhuffushi Regional Hospital, Kulhudhuffushi City, MDV; 2 Emergency Department, Kulhudhuffushi Regional Hospital, Kulhudhuffushi City, MDV

**Keywords:** cognitive impairment, elderly patient care, gait disturbance, normal pressure hydrocephalus, urinary incontinence (ui)

## Abstract

Normal pressure hydrocephalus (NPH) is a potentially reversible neurological condition in older adults characterized by gait disturbance, cognitive decline, and urinary incontinence, but its diagnosis is frequently delayed or missed, especially when patients present with other acute medical issues. We report two illustrative cases where probable NPH was incidentally detected during hospitalization for unrelated acute presentations. In both instances, brain imaging performed for other diagnostic purposes revealed ventriculomegaly, and subsequent cerebrospinal fluid (CSF) analysis confirmed normal opening pressure and composition. Further history revealed previously unaddressed symptoms consistent with probable NPH. Although surgical intervention was not pursued due to advanced age, comorbidities, and patient or family preferences, the diagnosis allowed for informed decision-making and supportive care planning.

These cases highlight how NPH may remain cryptic in elderly patients, especially when acute, self-limiting disorders dominate the clinical picture. They underscore the importance of maintaining a broad differential diagnosis and obtaining neuroimaging when chronic symptoms such as urinary incontinence, gait impairment, or cognitive decline are present, even when overshadowed by other diagnoses. Given that timely shunting can result in marked improvements in mobility, cognition, and bladder control in many patients, early identification is essential. At the same time, management of NPH must be individualized, taking into account patient age, comorbidities, surgical risks, and personal or family preferences. In both cases, while definitive intervention was not pursued, awareness of the diagnosis enabled informed shared decision-making and offered targeted long-term surveillance and supportive care.

## Introduction

Normal pressure hydrocephalus (NPH) is a subtype of communicating hydrocephalus characterized by ventriculomegaly without a corresponding increase in cerebrospinal fluid (CSF) pressure on lumbar puncture. First described by Hakim and Adams in the 1960s, NPH is classically associated with a clinical triad of gait disturbance, cognitive impairment, and urinary incontinence, commonly referred to as “wet, wobbly, and wacky” [1]. Importantly, NPH represents one of the few potentially reversible causes of dementia in older adults, underscoring the need for timely recognition and early intervention [2].

NPH is broadly categorized into two forms: idiopathic NPH (iNPH), characterized by the absence of an identifiable precipitating cause, and secondary NPH, which is associated with known etiologies such as traumatic brain injury, subarachnoid hemorrhage, central nervous system infections, tumors, or prior neurosurgical interventions [2]. The idiopathic form is more prevalent than commonly perceived. A population-based study from Western Sweden estimated a prevalence of 0.2% in individuals aged 70-79 years, rising to 5.9% in those aged 80 years and older [3]. Community-based studies in Japan report similar findings, with prevalence ranging from 1% to 2.9% among elderly populations [4]. Despite these figures, iNPH remains underdiagnosed, often due to its overlapping clinical features with other neurodegenerative and vascular disorders of aging, as well as frequent misattribution of symptoms to normal aging or more common dementias such as Alzheimer’s disease. Delayed diagnosis is common, with some studies reporting a median delay of over 12 months from symptom onset to diagnosis [2].

The underlying pathogenesis of NPH involves disturbed CSF dynamics within a communicating ventricular system. Proposed mechanisms include reduced subarachnoid space compliance, hyperdynamic CSF flow, impaired CSF absorption at the arachnoid granulations, and periventricular ischemia caused by chronic interstitial edema and ventricular stretch [5]. Disruptions in frontal-subcortical neural circuits in normal pressure hydrocephalus (NPH) primarily result in a classic triad of symptoms: gait apraxia due to the involvement of the periventricular corticospinal tracts, executive dysfunction from impaired frontostriatal connectivity, and urinary incontinence related to the compression of periventricular fibers involved in micturition control [5,6]. The diagnostic criteria are divided into three categories: possible, probable, and definite iNPH. Possible iNPH is diagnosed when one or more symptoms from the clinical triad are present, other potential causes are excluded, and there is no history of conditions known to cause secondary hydrocephalus. Probable iNPH is identified when the criteria for possible iNPH are met in addition to normal cerebrospinal fluid pressure and composition, along with either imaging features suggestive of disproportionately enlarged subarachnoid space hydrocephalus or clinical improvement following a cerebrospinal fluid tap test or drainage procedure. Definite iNPH is confirmed when the patient shows objective improvement in symptoms after cerebrospinal fluid shunt surgery [7]. The diagnosis of NPH requires a multimodal approach that includes clinical evaluation, cerebrospinal fluid (CSF) studies, and characteristic neuroimaging findings on magnetic resonance imaging (MRI). These imaging features typically include an Evans index greater than 0.3, enlargement of the temporal horns (more than 4 mm), a narrowed callosal angle (less than 90°), tight high-convexity sulci, and the presence of disproportionately enlarged subarachnoid space hydrocephalus (DESH), which is considered a hallmark of idiopathic NPH (iNPH) [8].

A normal opening pressure on lumbar puncture (<20 cm H₂O) is an important supportive finding in the evaluation of normal pressure hydrocephalus (NPH), as it aids in distinguishing NPH from other forms of hydrocephalus associated with elevated intracranial pressure. While not definitive in isolation, it contributes to the overall diagnostic framework when interpreted alongside clinical features and neuroimaging findings. Diagnostic certainty may be enhanced by large-volume CSF tap testing or extended lumbar drainage, both of which can result in transient but measurable improvement in gait or cognition and are predictive of positive response to shunting [9,10].

The treatment of choice for idiopathic normal pressure hydrocephalus (iNPH) is cerebrospinal fluid (CSF) diversion, most commonly via ventriculoperitoneal (VP) shunt placement. Outcomes are generally more favorable when diagnosis and intervention occur early in the disease course. Shunt surgery has been shown to produce symptomatic improvement across multiple domains. Among these, gait disturbance exhibits the highest rate of responsiveness, with improvement reported in approximately 70%-85% of cases. Cognitive function improves in an estimated 60%-80% of patients, while bladder dysfunction shows improvement in 50%-80% [8,11]. These outcomes underscore the potential benefits of timely intervention in appropriately selected individuals. A recent meta-analysis found that the overall efficacy of CSF diversion procedures for iNPH has remained stable at approximately 74% between 2005 and 2024. However, cognitive and urinary improvements were observed in only about half of the cases, and no single surgical technique demonstrated clear superiority. These findings highlight the ongoing need for improved diagnostic criteria and patient selection strategies to optimize outcomes [12].

Despite these promising outcomes, the decision to pursue surgical intervention must account for factors such as age, frailty, medical comorbidities, surgical risk, and patient or family preferences. Potential complications include shunt malfunction, subdural hematoma, and infection, necessitating a careful risk-benefit assessment [13].

NPH is frequently overlooked in clinical practice, particularly when elderly patients present with other acute neurological or systemic conditions. Misattribution of NPH symptoms to normal aging, delirium, or primary neurodegenerative disorders may delay diagnosis and appropriate management. In this report, we describe two elderly female patients, aged 92 and 75, whose acute presentations of hyponatremic encephalopathy and acute vestibular neuronitis initially obscured the underlying diagnosis of NPH. Incidental neuroimaging findings, along with supportive CSF analysis, led to the diagnosis of probable iNPH in both cases. Ventriculoperitoneal shunting was considered as a potential therapeutic option; however, conservative management was ultimately selected due to the patient’s advanced age and the presence of significant comorbidities, including hypertension, obesity, and advanced osteoarthritis of the knee. These factors were associated with an increased risk of perioperative complications. After a comprehensive discussion outlining the potential benefits and risks of surgical intervention, the patients and family members expressed a clear preference for non-surgical management, in keeping with a patient-centered approach to care.

These cases highlight the importance of maintaining a high index of suspicion for NPH, particularly when radiographic findings coincide with compatible clinical symptoms. Even when definitive surgical treatment is not pursued, recognizing the condition facilitates informed decision-making and enables appropriate counseling for patients and their families.

## Case presentation

Case 1: Hyponatremic encephalopathy and type II respiratory failure in a nonagenarian with incidental imaging findings compatible with probable normal pressure hydrocephalus

A 92-year-old woman with a history of long-standing hypertension and chronic immobility, bedridden for the past 15 years due to knee osteoarthritis, obesity, and aging, presented with a four-day history of progressively worsening consciousness. In the preceding week, she had experienced generalized weakness, reduced oral intake, and increased lethargy. There was no reported history of fever, seizure activity, recent head trauma, or focal neurological deficits. The family reported baseline mild cognitive impairment and a two-year history of urinary incontinence, which had been attributed to age-related decline and immobility.

On examination, she was obtunded with a Glasgow Coma Scale (GCS) score of 10 (E3, V3, M4) [14]. Formal cognitive assessment was not feasible due to fluctuating consciousness and premorbid functional status. Neurological examination revealed global hypotonia with preserved deep tendon reflexes and bilateral flexor plantar responses. Cranial nerve evaluation showed no focal abnormalities. Respiratory examination identified coarse crepitations at the right lung base.

Laboratory investigations revealed profound hyponatremia, with a serum sodium level of 113 mmol/L. Serum osmolality was 245 mOsm/kg. Urine studies demonstrated a sodium concentration of 111 mmol/L and an osmolality of 330 mOsm/kg, findings consistent with the syndrome of inappropriate antidiuretic hormone secretion (SIADH). The inflammatory marker C-reactive protein (CRP) was modestly elevated at 48 mg/L (reference range: 0-10 mg/L). Thyroid function tests and morning cortisol were normal. Other metabolic panels, including renal and liver function tests, complete blood count, and arterial blood gas, were within normal limits. The patient’s pertinent biochemical parameters are summarized in Table 1, providing an overview of electrolytes, osmolality studies, and endocrine evaluations.

**Table 1:** Summary of laboratory investigations of case 1 TSH: thyroid-stimulating hormone, T4: free thyroxine, T3: free triiodothyronine

Parameters	Values	Reference range
Serum sodium	113 mmol/L	135-145 mmol/L
Serum osmolality	245 mOsm/kg	275-295 mOsm/kg
Urine sodium	111 mmol/L	20-40 mmol/L
Urine osmolality	330 mOsm/kg	300-900 mOsm/kg
TSH	2 µIU/mL	0.47-4.94 µIU/mL
T4	14 pmol/L	10-28 pmol/L
T3	16.5 pmol/L	4.26-48.1 pmol/L
Morning cortisol (9 AM)	347 nmol/L	123-626 nmol/L

Given the presumed diagnosis of SIADH secondary to lower respiratory tract infection, she received cautious correction of serum sodium and appropriate antibiotic therapy. Her level of consciousness improved significantly.

However, shortly before planned discharge, she developed sudden-onset dyspnea and worsening confusion. Arterial blood gas analysis demonstrated type II respiratory failure with hypercapnia. Noninvasive ventilation (biphasic positive airway pressure (BiPAP)) was instituted for three days, successfully correcting CO₂ retention and improving respiratory function, although her altered mental status persisted.

**Figure 1:**
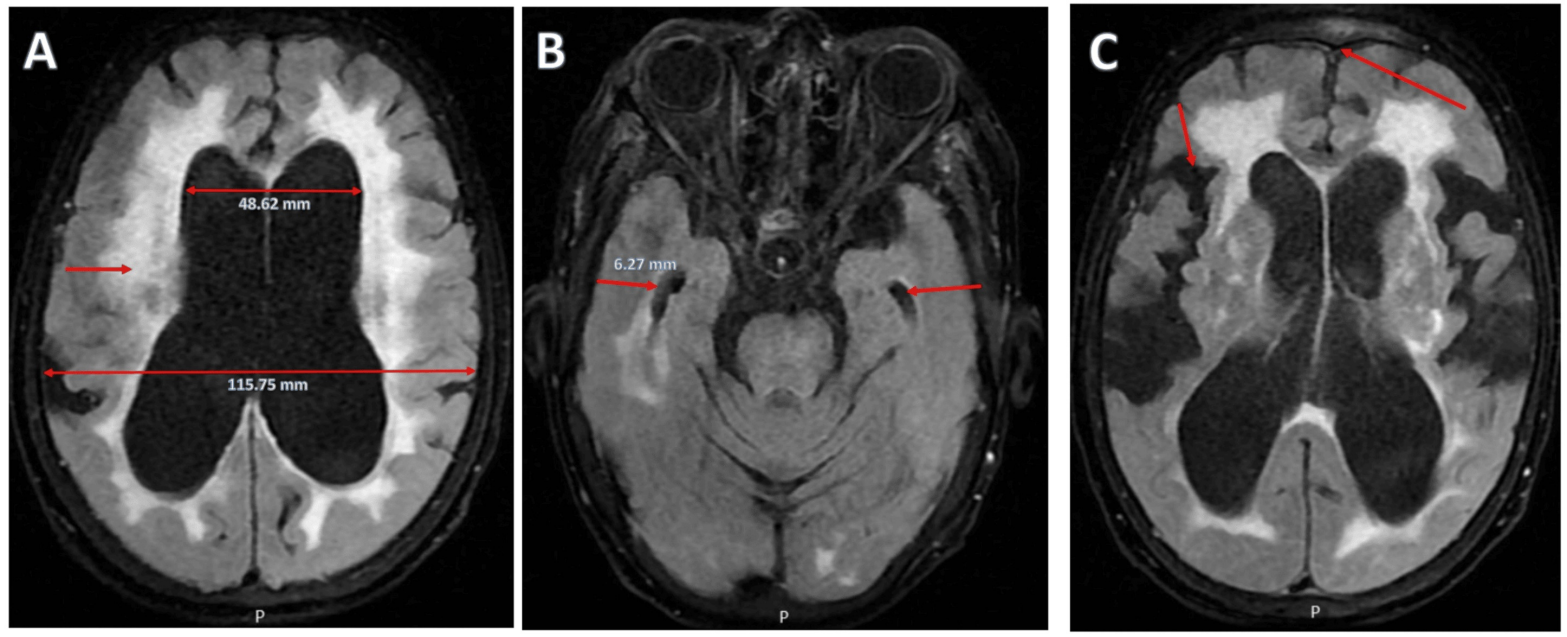
Brain MRI FLAIR sequences showing findings consistent with iNPH A: Axial FLAIR image demonstrating ventriculomegaly with increased transverse diameter of the lateral ventricles (double-headed arrows). Note the presence of symmetric periventricular hyperintensities adjacent to the ventricular margins (single arrow), consistent with transependymal CSF seepage. B: Axial FLAIR image at the level of the temporal lobes shows prominent temporal horns (arrows), a hallmark of ventricular dilation in iNPH. C: Axial FLAIR image highlighting DESH pattern, characterized by narrowed high-convexity sulci (long arrow) and enlarged Sylvian fissures (short arrow), supporting the diagnosis of iNPH. MRI: magnetic resonance imaging, FLAIR: fluid-attenuated inversion recovery, iNPH: idiopathic normal pressure hydrocephalus, CSF: cerebrospinal fluid, DESH: disproportionately enlarged subarachnoid space hydrocephalus

An MRI of the brain with contrast, performed to exclude osmotic demyelination or central pontine myelinolysis, revealed marked ventriculomegaly involving the lateral ventricles (Evans index: 0.42), enlarged temporal horn (width: 6.27 mm), periventricular hyperintensities, and disproportionately enlarged subarachnoid space hydrocephalus (DESH) (Figure 1).

Lumbar puncture revealed a normal opening pressure, and cerebrospinal fluid (CSF) analysis, including cell count, protein, and glucose levels, was within normal limits, as summarized in Table 2.

**Table 2:** CSF parameters of case 1 CSF: cerebrospinal fluid

CSF parameters	Values	Reference range
Opening pressure	18 cm H₂O	6-20 cm H₂O
Appearance	Clear	Clear, colorless
White blood cell count	2 cells/µL	0-5 cells/µL
Red blood cell count	0 cells/µL	0 cells/µL
Protein concentration	28 mg/dL	15-45 mg/dL
Glucose concentration	65 mg/dL	45-80 mg/dL (or 60%-70% of serum glucose)
Gram stain	Negative	Negative
Culture	No growth	No growth

A final diagnosis of acute SIADH-induced encephalopathy secondary to pneumonia, type II respiratory failure responsive to BiPAP, and incidental and suggestive of probable NPH was established.

Given the radiological and clinical evidence suggestive of NPH, along with a history of early urinary incontinence and mild cognitive impairment, it was considered that an underlying probable NPH process was unmasked by acute illness. The family was thoroughly counseled regarding ventriculoperitoneal shunting. Given the patient’s age, comorbidities, and family’s preferences, surgical intervention was declined. The patient was discharged with supportive care and outpatient follow-up in neurology and geriatrics. At outpatient follow-up, the patient continued to exhibit cognitive impairment and urinary symptoms. Following detailed discussions regarding the potential benefits and risks of further interventions, including repeated therapeutic lumbar punctures and ventriculoperitoneal (VP) shunt placement, the patient and family elected to decline invasive procedures and opted for conservative management, consisting of regular clinical monitoring through scheduled outpatient follow-up.

Case 2: Elderly woman with acute vestibular neuronitis unmasking normal pressure hydrocephalus

A 75-year-old woman presented with a two-day history of progressively worsening continuous vertigo, described as spinning, accompanied by nausea and vomiting. This symptom onset followed a recent upper respiratory tract infection characterized by fever, sore throat, and cough.

She denied hearing loss, ear discharge, head trauma, or seizures. Notably, she reported a four-year history of predominantly urge-type urinary incontinence and intermittent short-term memory lapses, which had not been previously evaluated.

On examination, the patient was alert and oriented. Cognitive assessment using the Mini-Mental State Examination (MMSE) revealed a score of 22/30, consistent with mild cognitive impairment [15]. Neurological assessment revealed a positive head impulse test on the left side, consistent with peripheral vestibular dysfunction. Unidirectional horizontal nystagmus was observed. Assessment for skew deviation was negative. There were no cerebellar signs such as dysmetria or dysdiadochokinesia. The Romberg test was negative with eyes closed, and the Dix-Hallpike maneuver did not provoke positional vertigo.

Otoscopy and audiologic reflex testing were unremarkable. Rinne and Weber tests were normal, excluding conductive or sensorineural hearing loss. Gait assessment demonstrated a slow, shuffling pattern with difficulty initiating steps, consistent with features of gait apraxia. The patient was unable to perform tandem walking, indicating impaired postural control. These findings are in keeping with the gait disturbance commonly observed in NPH and may also contribute to the patient’s subjective dizziness.

**Figure 2:**
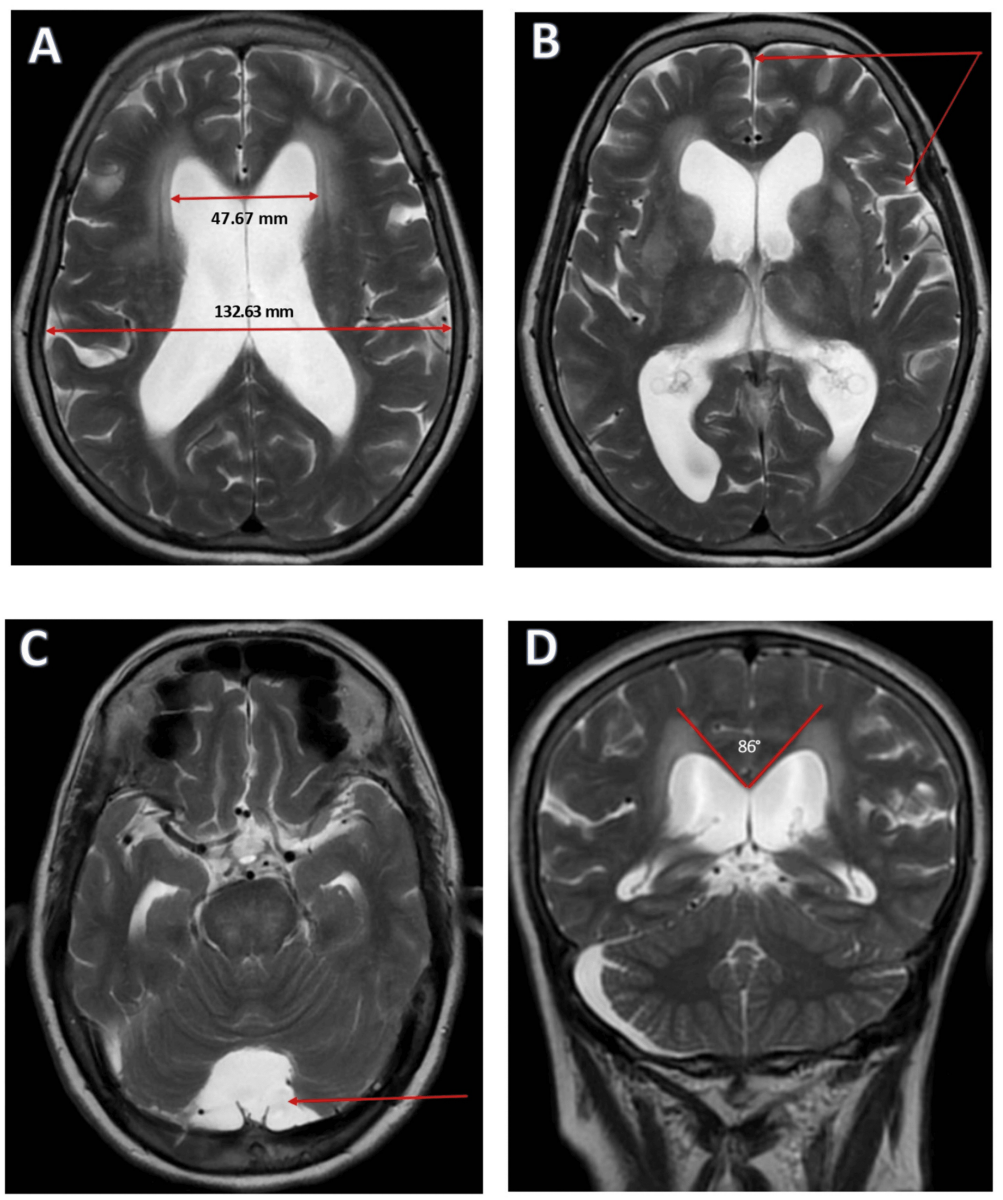
Brain MRI T2-weighted images showing features consistent with iNPH A: Axial T2-weighted image shows marked ventriculomegaly with increased Evans index (ratio of frontal horn width to maximal inner skull diameter, arrows), consistent with hydrocephalus. B: Axial T2 image demonstrating DESH pattern, with narrowing of high-convexity sulci and widened Sylvian fissures, a classic radiological feature of iNPH. C: Axial T2 image at the level of the posterior fossa reveals an enlarged fourth ventricle consistent with communicating hydrocephalus. D: Coronal T2-weighted MRI showing a narrow callosal angle (<90°), which is characteristic of iNPH. MRI: magnetic resonance imaging, iNPH: idiopathic normal pressure hydrocephalus, DESH: disproportionately enlarged subarachnoid space hydrocephalus

Laboratory investigations, including complete blood count, electrolytes, renal and liver function tests, thyroid profile, and vitamin B12 level, were within normal limits. Audiometry confirmed preserved hearing thresholds. Magnetic resonance imaging (MRI) of the brain revealed enlargement of the lateral and fourth ventricles, with an Evans index of 0.36. There was no evidence of obstructive lesions, and the measured callosal angle was 86° (Figure 2).

Lumbar puncture demonstrated a normal opening pressure, and cerebrospinal fluid (CSF) analysis was unremarkable, as detailed in Table 3.

**Table 3:** CSF parameters of case 2 CSF: cerebrospinal fluid

CSF parameters	Values	Reference range
Opening pressure	14 cm H₂O	6-20 cm H₂O
Appearance	Clear/colorless	Clear, colorless
White blood cell count	1 cells/µL	0-5 cells/µL
Red blood cell count	0 cells/µL	0 cells/µL
Protein concentration	32 mg/dL	15-45 mg/dL
Glucose concentration	62 mg/dL	45-80 mg/dL (or 60%-70% of serum glucose)
Gram stain	Negative	Negative
Culture	No growth	No growth

Based on the clinical history, imaging findings, and cerebrospinal fluid analysis, a dual diagnosis of acute vestibular neuronitis and incidental findings suggestive of probable NPH was established.

The patient was treated with vestibular suppressants (prochlorperazine) and initiated on vestibular rehabilitation therapy. Her vertigo resolved within one week. In light of the probable NPH diagnosis, the patient and her family were counseled regarding the potential benefits of ventriculoperitoneal shunting versus conservative management. Considering surgical risks and patient preference, conservative therapy was chosen, with plans for multidisciplinary outpatient follow-up involving otolaryngology, neurology, and geriatrics. At subsequent follow-up, the patient continued to exhibit mild cognitive impairment, gait abnormality, and urinary incontinence. In light of persistent symptoms and limited response to prior lumbar puncture, surgical intervention with VP shunting was recommended. However, the patient declined both further therapeutic lumbar drainage procedures and any operative intervention.

## Discussion

The two cases presented underscore the diagnostic challenge and clinical significance of recognizing normal pressure hydrocephalus (NPH) in elderly patients, particularly when it is incidentally discovered during evaluations for acute neurological or systemic illnesses. Both patients exhibited radiological ventriculomegaly and fulfilled clinical criteria consistent with probable NPH, yet their presentations were initially dominated by unrelated acute conditions, metabolic encephalopathy and vestibular neuronitis, respectively. These scenarios highlight the importance of maintaining a broad differential diagnosis in geriatric patients, especially when subtle or overlapping symptoms mimic other neurodegenerative or cerebrovascular disorders [1,2].

In the first case, a 92-year-old woman with subacute altered mental status and severe hyponatremia was initially managed for syndrome of inappropriate antidiuretic hormone secretion (SIADH) secondary to lower respiratory tract infection. Despite correction of metabolic derangements and resolution of infection, persistent cognitive impairment and gait disturbances prompted further evaluation. Brain MRI revealed ventriculomegaly, characteristic of probable NPH, confirmed by normal CSF opening pressure on lumbar puncture. This case illustrates how acute metabolic encephalopathy can unmask underlying probable NPH, which might have otherwise been overlooked in the context of premorbid cognitive decline and immobility [3,5,7,8].

The second patient, a 75-year-old woman who presented with acute vertigo secondary to vestibular neuronitis, had a pre-existing history of urinary incontinence and intermittent memory complaints. Neuroimaging revealed typical features of probable NPH, including enlarged lateral and fourth ventricles without obstructive pathology, and lumbar puncture supported by a normal cerebrospinal fluid (CSF) opening pressure (<20 cm H₂O) on lumbar puncture. The co-existence of acute peripheral vestibulopathy and incidental probable NPH in this patient highlights the complexity of diagnosing probable NPH in the presence of other neurological conditions and emphasizes the value of thorough clinical history and imaging [4,6,7,9].

Accurate diagnosis of NPH requires integration of clinical, radiological, and CSF findings. Both cases exhibited at least partial components of the classic triad-gait disturbance, cognitive decline, and urinary incontinence, along with supportive MRI findings such as ventriculomegaly (Evans index: >0.3), enlarged temporal horns, and disproportionately enlarged subarachnoid space hydrocephalus (DESH) pattern, in the absence of obstructive hydrocephalus [8,10]. Normal CSF opening pressures further corroborated the diagnosis of probable idiopathic NPH, aligning with current diagnostic guidelines [7,11].

These cases carry several important clinical implications. First, NPH remains underdiagnosed and often misattributed to “normal aging” or irreversible dementia, particularly in elderly patients with multiple comorbidities. Clinicians should maintain heightened vigilance for NPH in older adults presenting with gait instability, urinary symptoms, or cognitive impairment, especially when these features evolve insidiously or coexist with acute medical conditions [1,7,8]. Second, acute illnesses may precipitate clinical deterioration or unmask previously compensated NPH, as seen in these reports. Third, early recognition is paramount since NPH represents one of the few potentially reversible causes of dementia and gait dysfunction in the elderly, with ventriculoperitoneal shunting yielding improvement in 60%-85% of carefully selected patients [8,11,12]. Lastly, shared decision-making is critical; patient age, frailty, comorbidities, surgical risks, and patient/family preferences must guide individualized management [12,13]. Both patients’ families opted for conservative management after detailed counseling on risks and benefits, reflecting real-world challenges in treating frail elderly patients [11,12].

## Conclusions

These cases highlight the importance of maintaining clinical vigilance when incidental ventriculomegaly is identified in elderly patients, particularly those presenting with acute neurological or systemic conditions. Normal pressure hydrocephalus (NPH) is frequently underdiagnosed, with its hallmark features, such as gait disturbance, cognitive impairment, and urinary incontinence, often misattributed to other geriatric syndromes. Failure to consider NPH in the differential diagnosis may result in missed opportunities for treating a reversible cause of functional decline.

A structured diagnostic approach involving neurological assessment, neuroimaging, and cerebrospinal fluid (CSF) analysis is essential for confirming the diagnosis. While CSF diversion is the standard intervention and can lead to significant improvements in mobility, cognition, and continence, conservative management may be appropriate in selected patients, particularly those with advanced age, significant comorbidities, and individual preferences. These cases underscore the need for early recognition and individualized treatment strategies, even in the absence of surgical intervention.
